# Strain Measurements of Composite Laminates with Embedded Fibre Bragg Gratings: Criticism and Opportunities for Research

**DOI:** 10.3390/s110100384

**Published:** 2010-12-31

**Authors:** Geert Luyckx, Eli Voet, Nicolas Lammens, Joris Degrieck

**Affiliations:** Department of Material Science and Engineering, Ghent University, Sint-Pietersnieuwstraat 41, 9000 Ghent, Belgium; E-Mails: Eli.Voet@UGent.be (E.V.); Nicolas.Lammens@UGent.be (N.L.); Joris.Degrieck@UGent.be (J.D.)

**Keywords:** fibre Bragg grating, embedded, strain measurements, composite materials

## Abstract

Embedded optical fibre sensors are considered for structural health monitoring purposes in numerous applications. In fibre reinforced plastics, embedded fibre Bragg gratings are found to be one of the most popular and reliable solutions for strain monitoring. Despite of their growing popularity, users should keep in mind their shortcomings, many of which are associated with the embedding process. This review paper starts with an overview of some of the technical issues to be considered when embedding fibre optics in fibrous composite materials. Next, a monitoring scheme is introduced which shows the different steps necessary to relate the output of an embedded FBG to the strain of the structure in which it is embedded. Each step of the process has already been addressed separately in literature without considering the complete cycle, from embedding of the sensor to the internal strain measurement of the structure. This review paper summarizes the work reported in literature and tries to fit it into the big picture of internal strain measurements with embedded fibre Bragg gratings. The last part of the paper focuses on temperature compensation methods which should not be ignored in terms of *in-situ* measurement of strains with fibre Bragg gratings. Throughout the paper criticism is given where appropriate, which should be regarded as opportunities for future research.

## 1. Introduction

### 1.1. Composite Structures

There is a growing interest in the use of fibre reinforced plastics (FRPs) as high-grade construction materials that need to be lightweight, yet strong under sometimes harsh loading conditions, for various applications [1–3]. Despite the growing popularity of structural composite materials, one has to realize that their mechanical behaviour is significantly different compared to conventional isotropic construction materials. The feedback from recorded loads, deformations and temperatures of (and especially inside) existing structures in real conditions, can lead to highly valuable information for design criteria. In particular, strain monitoring of an in-service structure should greatly enhance the insight and confidence in the (long-term) behaviour of high performance composite structures.

Therefore, research is being conducted worldwide on evaluation techniques to measure strain of different types of materials and structures. A major field of research concerns the application of optical fibre sensors [4–7], which have a number of well-known advantages including insensitivity to electromagnetic interference, small dimensions, light weight, multiplexing capabilities and resistance to corrosion [8]. The compatibility of these sensors with the manufacturing process of fibrous composite materials (e.g., pultrusion [9], co-braided with braided composites [10–13], laminates [14], *etc.*) can be seen as an extra advantage.

Within the group of the embedded optical fibre sensors, fibre Bragg gratings (FBGs) are the most widespread for measuring the internal deformations of various types of fibrous composite structures. By integrating an FBG into a structure, it becomes very robust and it can survive the sometimes harsh environment in which FRPs are used.

### 1.2. Technical Issues Concerning Embedding FBGs in Composite Materials

Despite the many advantages of embedded optical fibre sensors, integrating them into structures leads to a number of specific problems which need to be tackled. A major issue is the *entry point of the optical fibre* lead in the composite material, which is prone to breaking. An overview of the literature on approaches to overcome this problem is given by Green *et al.* [15]. Two main options exist to protect the fibre egress point in composite laminates: either integrating a fibre connector at the edge or surface of the laminate [16] or integration of a fibre feed-through mechanism [17]. Both methods enable the optical fibre to be led smoothly out of the stiff laminate (surface or edge) without excessive bending and curvature. However, decades of research have enabled the research community to come up with the ideal entry point in a material which is insensitive to delamination. Perhaps one should focus on eliminating the entry point entirely by wireless transmission of data from the embedded sensor to a read-out unit. On this subject, Teitelbaum *et al.* focused on wireless transmission of video data via a multimode fibre in a smart structure [18,19]. In SMARTFIBER, a European FP7-project, the researchers try to miniaturize the read-out unit, so that it can be embedded in the composite material [20]. With the last two solutions, edge trimming, which is commonly needed after composite fabrication, will become possible.

**Figure 1.**
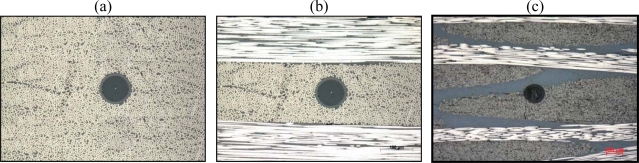
FBG embedded **(a)** in a unidirectional laminate, **(b)** in a cross-ply laminate, **(c)** in a cross-ply woven fabric laminate.

Another issue is *the distortion of the composite structure* in the surroundings of the optical fibre. The size of the FBG sensor ranges from 125 μm (which is a classical telecom fibre) down to 52 μm [21]. This is still one order of magnitude larger than the most commonly used reinforcement fibres (glass: 5–20 μm, carbon: 5–10 μm). Thus, the embedded optical fibre will inevitably cause a local distortion in the host material (Figure 1).

Minimizing the optical fibres will reduce the composite distortion. However, not only the mismatch in size between the optical fibres and the reinforcement fibres, but also the type of composite material which is used (uni-directional, woven fabric, stitched, braided, *etc*.), and the relative alignment of the optical fibre with respect to the reinforcement fibres influences the distortion. Research has proven that small diameter optical fibres do not cause any significant reduction in strength of composites and standard 125 μm optical fibres produce a minimum perturbation of the host material when embedded parallel to the reinforcing fibres in laminates [22,23]. In addition, for small diameter optical fibres, no resin-rich regions (also called resin pocket) are found around the fibre which to the contrary in some cases, can be found for embedded standard telecom optical fibres [24]. Shivakumar and Emmanwori embedded optical fibres with a relative orientation to the reinforcement fibres and found that the structural properties were not affected when the fibre was embedded parallel to the reinforcement fibres [25].

### 1.3. Strain Measurement Technique with Embedded FBG

The above mentioned structural strength analysis does not consider the *disturbance of the strain field in the composite material*. The strain field around an embedded optical fibre is significantly changed and the interfacial stresses are increased [26]. In addition, the stress/strain field present in the composite host material will differ significantly from the one present in the core of the optical fibre, due to the different mechanical properties of both materials. Several authors have accepted the challenge of measuring strains with FBGs embedded in several types of composite material for axial as well as multi-axial strain measurements. Even cure monitoring using embedded FBGs has been tackled in the literature. This review paper gives an overview of the methods to measure strain in composite materials with embedded fibre Bragg gratings and of the problems which needed to be tackled. Wherever necessary, critical notes will be made concerning the referred papers which should be seen as an opportunity for future research. It should be mentioned that the focus is on strain measurements and less on direct damage detection, though some reference to the authors concerned may occur. The paper starts by giving a short introduction on the basic principle of FBGs and how they are used to measure external strain and temperature influences.

## 2. Basic FBG Principle

A fibre Bragg grating is a *local modulation of the refractive index* in the optical fibre which can be created by an appropriate sideways illumination with UV-light (spatial fringe pattern) of a photo-sensitive optical fibre. This pattern in the core of the optical fibre can be created by using a holographic method (first introduced by Meltz *et al.* [27]) or a phase mask method. Both techniques can also be combined [28]. A point-by-point technique using a pulsed laser (each pulse inscribes one grating period) can be used as well. This technique is mainly used to inscribe long period gratings [29]. Although several grating types (uniform, chirped, apodized, *etc*.) exist, *uniform FBGs* will be the main focus of this review paper. When light with a broadband spectrum is coupled into a single mode (SM) optical fibre and interacts with the grating, only a small part of the light spectrum will be back reflected. The reflected spectrum is centred on *the Bragg-wavelength* (*λ*_B_) and depends on the *effective index of refraction* (*n*_eff_) and on the *Bragg period* (Λ) of the grating according to the well known Bragg equation:

(1) $$\lambda_{B}=2n_{\text{eff}}\Lambda$$

By inscribing several FBGs with different grating periods in the same optical fibre, an array of gratings can be manufactured. This allows the user to monitor different positions in the structure with only one sensor line. *Both the effective refractive index and the Bragg period of the grating will be affected by any applied mechanical strain as well as by temperature.*

### 2.1. Strain Sensitivity

**Figure 2.**
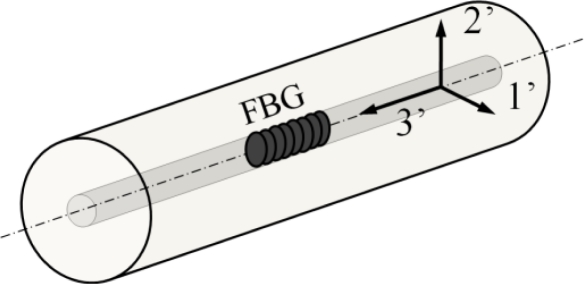
The used coordinate system of an optical fibre.

In most cases, for FBGs written in conventional single mode optical fibres, the centre strain approximation can be used to determine the theoretical strain and temperature response [30]. Under this approximation, each Bragg peak wavelength (corresponding with the 1’- and 2’-axis, Figure 2) is dependent on the total strain field present in the core of the optical fibre sensor. Under the assumption of an *isothermal condition* (Δ*T* = 0) the wavelength shifts for small strain perturbations can be written as:

(2) $$\begin{array}{l} \frac{{\Delta \lambda }_{B,1\prime }}{\lambda_{B,1\prime }}=\varepsilon_{3\prime }-\frac{1}{2}n_{\text{eff},1\prime }^{2}\;\left[p_{11}\varepsilon_{1\prime }+p_{12}\;\left(\varepsilon_{2\prime }+\varepsilon_{3\prime }\right)\right]\\ \frac{{\Delta \lambda }_{B,2\prime }}{\lambda_{B,2\prime }}=\varepsilon_{3\prime }-\frac{1}{2}n_{\text{eff},2\prime }^{2}\;[p_{11}\varepsilon_{2\prime }+p_{12}\;\left(\varepsilon_{1\prime }+\varepsilon_{3\prime }\right)] \end{array}$$

where ε_1′_ε_2′_ε_3′_ are the *principal strain components* along the axes of the fibre’s coordinate system (Figure 2). The ′ is used to avoid confusion with the numbers used to point out the different gratings in a sensor array (e.g., for temperature compensation, Section 4). The strain components ε_4′_ ε_5′_ and ε_6′_ are usually neglected in terms of the sensor response [31,32]. Further in Equation 2, Δ*λ_B_* is the *Bragg peak wavelength shift*, *λ*_B_ the *initial mean Bragg peak wavelength*, *p*_11_ and *p*_12_ are the *strain-optic coefficients*. It is clear from Equation (2) that axial elongation (ε_3′_ > 0, ε_1′_ = ε_2′_ = −*υ*ε_3′_) or uniform compression (ε_1′_ = ε_2′_ < 0) will cause the Bragg peak reflection to shift to higher or lower wavelengths, respectively.

### 2.2. Temperature Sensitivity

Under the assumption of a *strain free condition* (*ε_i_* = 0) the Bragg peak shift under relatively small temperature load can be written as:

(3) $${\Delta \lambda }_{B}=2\left(n_{\mathrm{eff}}\;\frac{\partial \Lambda }{\partial T}+\Lambda \frac{{\partial n}_{\mathrm{eff}}}{\partial T}\right)\Delta T$$

Equation (3) represents the effects of temperature on the Bragg wavelength. A temperature increase causes a thermal expansion of the fibre with Bragg grating (and thus a change of its Bragg period) and also a change in the refractive index. This fractional wavelength shift for a small temperature change Δ*T* may be written as [33]:

(4) $$\begin{array}{ll} {\Delta \lambda }_{B} & =\lambda_{B}\left(\alpha_{f}+\alpha_{n}\right)\Delta T\\ & \;\;=\lambda_{B}\;\beta \Delta T \end{array}$$

where $\alpha_{f}=\frac{1}{\Lambda }\frac{\partial \Lambda }{\partial T}$ is the *thermal expansion coefficient* of the optical fibre (approximately 0.55 × 10^−6^ 1/K for silica [34]). The quantity $\alpha_{n}=\frac{1}{n_{\text{eff}}}\frac{{\partial n}_{\text{eff}}}{\partial T}$ represents the so-called *thermo-optic coefficient*, which is dependent on the type and concentration of dopant(s). Values between 3.0 × 10^−6^ [35] and 8.6 × 10^−6^ 1/K for a germanium-doped, silica-core fibre have been reported [8]. The coefficients *α_f_* and *α_n_* can be combined in the so-called *temperature coefficient β*. Clearly the index change is by far the dominant effect. From Equation (4), it can be calculated that the linear temperature sensitivity of an FBG with a thermo-optic coefficient of 5.9 × 10^−6^ 1/K [36] in the C-band (e.g., 1,550 nm) is ∼10 pm/K.

In many cases bigger temperature variations are to be expected (e.g., in cure monitoring of FRPs). Therefore, one cannot rely on the linear relation of Equation (4) anymore. Note that the expansion coefficient *α_f_* is constant over a high temperature range [37], however, the thermo-optic coefficient *α_n_* is *temperature dependent* (*α_n_* = *αT* + *b*, [38]). Therefore, when considering high temperature ranges, a more accurate equation is necessary. By substituting this linear dependency in Equation (4), it can be rewritten as:

(5) $${d\lambda }_{B}=\lambda_{B}\;\left(\alpha_{f}+(\mathrm{aT}+b)\right)\mathrm{dT}$$

**Figure 3.**
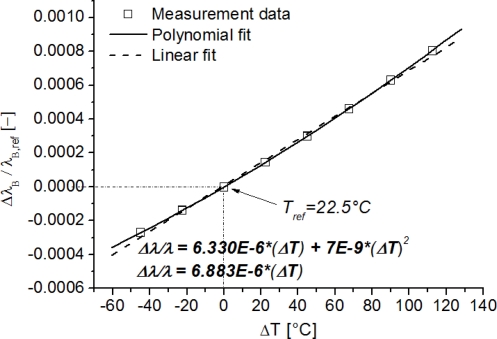
Temperature calibration (ranging from −22.5 °C–135 °C) of an FBG written in a Ge-doped optical fibre.

Pal *et al.* [39] already fitted data of temperature calibrations of fibre Bragg gratings to higher order polynomials and found satisfying results for a *quadratic polynomial*. An example of typical non-linear effects in the temperature calibration curve of an FBG written in a Ge-doped optical fibre is shown in Figure 3. Here, the normalized wavelength shift Δ*λ_B_*/*λ_B_* is plotted against the temperature shift *ΔT*, with respect to a reference temperature (e.g., T_ref_ = 22.5 °C). If a linear regression is used instead of a polynomial (Figure 3), large errors can be made if used when compensating strain measurements for temperature fluctuations, in for example real-life applications. For a temperature change of 120 °C, a wavelength shift error of approximately 60 pm or a longitudinal strain error of approximately 70 μstrain is introduced.

It should be apparent that any change in wavelength, associated with the action of an external perturbation to the grating, is the *sum of mechanical deformation and temperature terms*. Therefore, in sensing applications where only one perturbation is of interest, the de-convolution of temperature and strain becomes necessary! An overview of methods to decouple the temperature and strain cross sensitivity is given in Section 4, however, focus will first be on pure strain measurements.

## 3. Strain Measurements with Embedded FBGs

**Figure 4.**
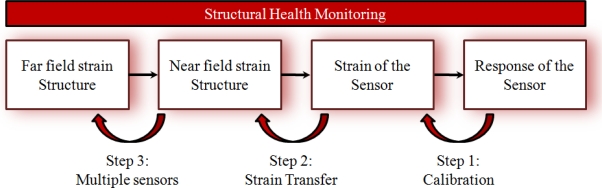
Flow chart of the different steps necessary for structural health monitoring using an embedded optical fibre sensor [40].

The monitoring scheme of Figure 4 gives an overview of the different steps necessary to relate the output of an embedded FBG to the strain of the structure in which it is embedded. Each step has been addressed in literature, and an overview can be found in this review paper.

When going from right to left in this monitoring scheme, *the response of the sensor* (Bragg wavelength shifts) should be related to *the strain of the sensor* (longitudinal and/or transversal). This requires a basic (theoretical and experimental) know-how on the strain dependency of FBGs written in optical fibres (step 1, calibration). The shape of the cured spectrum can play a significant role in this (Section 3.1). Secondly, since the sensor (silica glass optical fibre) and the host material (fibre reinforced plastic) have different material properties, the mechanical interaction between the embedded sensor and its host should be evaluated (this is step 2, strain transfer relationship) to determine the *near field strain in the structure*. In the third and final step, the strain at some discrete positions in the structure can be used as a measure for its global condition (step 3, multiple sensors), *i.e.*, the *far field strain in the structure*. In this paper the authors focus on the first two steps in the scheme.

### 3.1. FBG Sensor Response

#### 3.1.1. Calibration of a Non-Embedded FBG

Calibration of a non-embedded FBG, or step 1 (Figure 4) has been extensively addressed in the literature without considering the embedded situation. It should be subdivided in *longitudinal strain* and *transversal strain* calibrations. Longitudinal strain calibrations of FBGs are more or less straight-forward and therefore, only a few research results are to be reported. For example Abe *et al.* [41] fixed the fibre between two displacement stages, where a known deformation can be applied by a calibrated micrometer. The longitudinal strain sensitivity of an internal elliptical cladding fibre was determined. One should, however, mention that this method is not suited for locally stripped fibres. The strain will vary significantly when comparing a coated section in the fibre with the locally stripped fibre section. Hence, large errors are induced when determining the strain sensitivity. To determine the strain sensitivity of stripped FBGs, one needs to measure the applied load during calibration and relate that to the stress/strain in the fibre.

**Figure 5.**
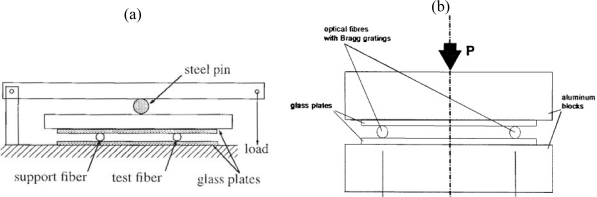
**(a)** Calibration device using a leverage arm to load the FBG and the support fibre [30]. **(b)** Calibration device using a load stamp to simultaneously load two FBGs [32].

When transversal strains are envisaged a different approach is necessary. Generally, a diametrical compression test set-up is used for transversal strain calibrations (Figure 5). By applying a diametric load on an axi-symmetric optical fibre in which an FBG is inscribed, a well known state of plane strain (ε_3′_ = 0) is produced [42]. Then, by substituting this strain state in Equation (2) the strain-optic coefficients can be determined experimentally.

In the literature several designs for transverse strain calibrations are proposed, which can be subdivided into two main groups. The first group of authors uses a leverage arm and calibrated weights to load the fibre [30,43–46], the other group directly press the fibre with calibrated weights or with a test bench [32,42,47–50] (Figure 5). In the first group, Abe *et al.* [44] used two support fibres and one test fibre to quantify the effect of transverse loads on the Bragg spectrum. Two support fibres can, however, compromise the measurement, since, the load should be exactly on top of the test fibre and all fibres should have exactly the same diameter. Therefore, Wagreich *et al.* [43] used only one support fibre. Later, Lawrence *et al.* [30] and Bosia *et al.* [45] improved the test set-up by reducing the number of steel pins. The resulting test set-up is shown in Figure 5(a). In the second group, the differences amongst the different test set-ups are mainly related to the use of guiding pins of the load stamp [47,48] or not [32,49,50]. By removing the guiding pins, all friction is eliminated out of the system, however, when the load stamp moves during calibration, the orientation of the optical fibre (certainly for polarization maintaining fibres (PM-fibres)) becomes uncertain.

Both methods are mostly used for calibration of FBGs written in high birefringent fibres (HiBi-fibres). Often the obtained Bragg shifts are only related with the diametrical applied force (in Nmm^−1^) and not with the necessary induced strain field in the core of the fibre. Stress applying parts in such HiBi-fibres make it difficult to predict those strains field. Therefore, it is far from easy to define the exact strain-optic coefficients of such fibres [45]. In addition, in most of these set-ups the researchers use a single FBG and a dummy support fibre to spread the applied load. Guemes and Menendez [32] were the first to suggest the use of two FBGs to improve the accuracy of the calibration set-up. As such, averaging possible asymmetries due to misalignments in the applied loads can be done. Voet *et al.* [50] used the same method and found little variation in the strain-optic coefficients for draw tower gratings (DTG®s) compared to what Bertholds and Dändliker found for standard telecom fibre [51]. DTG®s are single axial fibre Bragg gratings, written in highly GeO_2_-doped silica fibre using a single laser shot, during the fibre drawing process [52].

#### 3.1.2. FBG Spectral Response after Embedding

Dependent on the composite morphology, in which the FBG is embedded and the coating of the FBG, the reflected spectrum of the Bragg grating (after curing) can remain *uniform, specifically distorted, or highly randomly distorted*. Distortion of the FBG spectrum is mainly caused by the existence of *residual strains*, which build up during the composite manufacturing process. These strains exist in the absence of any external load (mechanical, and thermal).

On the microscopic level they arise from the mismatch in material properties between the (stiff) reinforcement fibres and the (soft) resin. On macroscopic level, they can arise from ply anisotropy (e.g., non balanced, non symmetric laminate). For composite structures in general, these strains play a significant role in their future mechanical performance.

**Figure 6.**
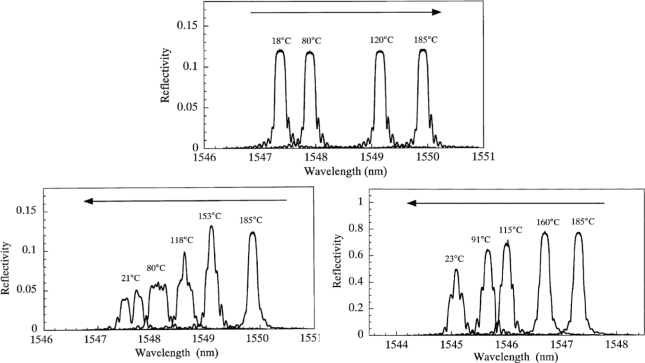
Reflection spectra from an FBG sensor, which was embedded into a CFRP laminate, measured during the cure cycle. (top) During the heating process and (bottom) during the cooling process, (bottom left) for an uncoated sensor, (bottom right) for a coated sensor [53].

In the literature, the *initiation and growth of spectral distortion* has been addressed, and some examples are given. Guemes and Menéndez [32] embedded two uni-axial FBGs in a carbon/epoxy laminate with a quasi-isotropic configuration ([45,−45,0,90,0,−45,45,0,90,45,−45]_2s_). The fibres were embedded in between two intermediate plies parallel to the reinforcing fibres. They observed that the shape of the spectrum remained unaltered during the heating process. However, at the beginning of the cooling process, the residual strains promoted by the thermal contraction appear, which affect the spectrum and even lead to a splitting of the Bragg peak. This splitting indicates that a certain transverse strain component (Δε_1′_−Δε_2′_) has developed during the curing. Okabe *et al.* [53] compared the influence of the occurring residual curing strain on uncoated, polyimide coated normal and polyimide coated small diameter FBG sensors. The fibres were embedded in a cross ply laminate [0_2_,90_4_,0_2_] in the 0 degree ply, in contact with the 90 degree ply. During the heating process, the shape of the spectrum of all sensors remained unaltered (Figure 6, top). During the cooling process, for the uncoated FBG sensor, birefringence effects are induced in the optical fibre, leading to a spectrum with two distinct peaks (Figure 6, bottom left). This spectral splitting was not observed for the embedded polyimide coated FBGs (Figure 6, bottom right), which indicates that a coated fibre exhibits a kind of “buffering” effect for transversal strains. Kim *et al.* [54] embedded an array of FBG sensors in a carbon laminate [0,45,90,−45]_2s_. During curing, a broadening of the spectrum could be observed, which indicates the occurrence of transverse stress.

**Figure 7.**
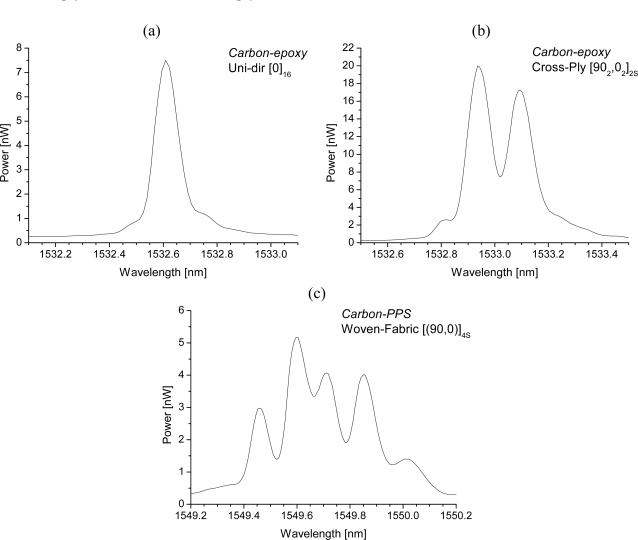
Spectrum of an uncoated FBG embedded **(a)** in a unidirectional laminate, **(b)** in a cross-ply laminate, **(c)** in a cross-ply woven fabric laminate.

It should be clear that every type of distortion needs a different approach in determining the strain components of the structure in which the sensor is embedded. Examples of the spectral response of embedded FBGs in different FRP laminates, is shown in Figure 7. An overview of the possible methods to interpret these spectra is given in the next section.

#### 3.1.3. Bragg Wavelength Determination

The interpretation of the spectrum in Figure 7(a) is straightforward. During a longitudinal calibration, the shift of the spectrum can directly be linked with the gauge factor of the FBG. Several methods are reported to analyze the shift of such a spectrum of which the ‘Full Width at Half Maximum’ (FWHM) algorithm and the ‘centre of gravity’ (COG), or ‘centroid’ algorithm are the most popular ones [55,56].

We note that the measurement of the Bragg-wavelength first of all depends on the stability and reproducibility of the employed interrogators. The measurement algorithm of the interrogator, however, is as important. The determination of the Bragg wavelength using a FWHM algorithm can differ from a Bragg wavelength determined by a centroid calculation algorithm. This is due to the influence of the grating characteristics (mainly uniformity of the spectral shape, amplitude) and the sampling density of the FBG spectrum, as well as on the uncertainty of the curve fit algorithm for the determination of the Bragg wavelength. An overview of other demodulation techniques, using for example tuneable filters, are reported in Zhao *et al.* [57].

For the second spectrum [Figure 7(b)], the occurring residual strains during the production process of a carbon-epoxy cross-ply laminate causes the spectrum to split into *two well-separated Bragg peaks* one for each propagation mode. It should be mentioned that the FBG used in this example is non-coated and thus not buffered from the transversal strains present in the material. As a consequence, in most loading cases, the wavelength shift of light travelling according to both eigenaxes (*1′* and *2′*) will differ because of a different change of the refractive index induced by the strain field present in the core of the optical fibre [Equation (2)]. Both Bragg peaks can then be monitored separately (FWHM- or COG-algorithm). By adding one [30,41,44,58–60], or several [61,62] extra FBGs, one can think of making a multi-axial strain sensor. For example, Lawrence *et al.* [30], and Mawatari and Nelson [58] suggest inscribing an extra grating at the position of the first grating in a PM-fibre. To obtain a working solution, the wavelength of both Bragg sensors should be chosen sufficiently separated (for example, in the 1,300 nm and 1,550 nm range). A drawback of this solution is the need for two separate light sources. In Luyckx *et al.* [60] this drawback is overcome by encapsulating the second grating in a capillary. As such, the second grating is isolated from transverse stresses and will react differently to external loading of the composite structure than the first grating. The capillary will, however, locally cause more distortion to the composite. In most of these research papers the suggested sensors are only calibrated without considering embedment in composite structures.

**Figure 8.**
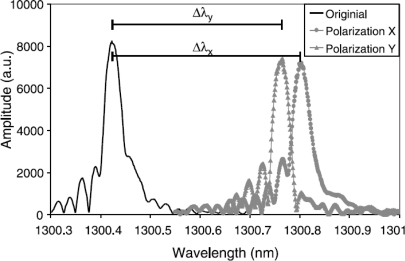
Spectral measurements of an embedded FBG before and after curing, using a tuneable laser with polarization control. The two right-hand peaks represent the two major polarization axes [69].

FBGs can be considered as a point sensor. However, local strain gradients (axial [63,64] as well as transversal strain [53,65–67]) can severely distort the Bragg spectrum. The difference between both is often difficult to make. A method to discriminate between both types can be found in the polarization control of the input light sent in the optical fibre [68,69] (Figure 8). Apart from this, the gradients can be related to the embedding process [70,71] of the FBG in the composite material or can originate from a specific loading case (e.g., bending [64,72]). For example Figure 7(c) represents the spectrum of an FBG embedded in a 5-harness satin weave thermoplastic composite. The axial strain profile of such a material has been numerically determined by Daggumati *et al.* [73]. The authors found that a clear strain gradient exist over the length of the grating and that matching axial strain results were found between the numerically determined profiles and measured profile. Kang *et al.* studied the response of a grating mounted on a beam which is clamped at one side and bended at the other [64]. In this way, a strain gradient is created over the length of the beam and the grating. One of the conclusions in this research is keeping the grating length as short as possible to minimize strain gradients over the grating. One should however remark that when embedded and measuring only the local strain over a short gauge distance inside a fibrous composite structure, this is not necessarily equal to the global strain of the structure [73–75].

Black *et al.* and Wang *et al.* characterized high birefringent fibres subjected to non-homogeneous transverse loads [65,76]. Under these non-homogeneous loads one of the peaks gets heavily distorted. Ling *et al.* simulated the reflection spectra of FBGs using the T-matrix formulation for a linear and a quadratic strain gradient [77]. This was done for small as well as for large strain gradients. Peters *et al.* presented a experimental verification of the response of embedded FBGs in epoxy specimens to applied non-homogeneous strain fields [78]. These strain fields could be controlled by machining the cross section of the specimen.

It is certain, that for *heavily distorted FBG spectra*, a simple demodulation technique such as FWHM-algorithm will not satisfy the need to quantify the global strain of a structure under load. Therefore, certain new techniques are necessary to predict the strain in a structure, and perhaps other parameters of the grating could be measured simultaneously.

A spectrum of an FBG written in a multimode optical fibre typically consists of more than one peak (dependent on the reflected modes). This forced Lim *et al.* to develop a cross-correlation algorithm to quantify the variation of wavelength shift caused by altering strain or temperature [79]. Caucheteur *et al.* developed a similar demodulation technique which evaluates the position of the reflection spectrum at the measurand (in this case temperature) with respect to the spectrum of an undisturbed sensor in the specific case of a twin Bragg grating [80]. One should note that for measurements subjected to large strain gradients a relative modulation technique will be more reliable.

### 3.2. Strain Transfer between Optical Fibre Sensor and Host Material

In the *strain transfer step* (step 2, Figure 4), a relation has to be found between the measured strain in the optical fibre and the one in the composite material. Beforehand, a clear difference should be made between uni-axial and multi-axial strain sensing.

#### 3.2.1. Uni-Axial Strain Sensing Applications

For *uni-axial strain sensing* applications, the optical fibre is usually provided with a protective polymer layer, *i.e.*, fibre coating. If one considers an embedded optical fibre, the coating will act as the interface between the optical fibre and the host material. It is clear that this can have a definite impact on the transfer of strains from the matrix to the fibre and by choosing the right coating, the strain transfer can be improved in certain principal directions. For example, it is possible to choose a coating for which stress concentrations around the fibre can be avoided and composite distortion minimize [81,82]. A lot of research on strain transfer mechanisms is related to surface mounted FBGs. One should, however, remark that the relation of the strain of the sensor to that of the substrate for surface mounted FBG strain sensors, embedded in a thin layer of adhesive, and bonded to the surface of a structure is substantially different from that of embedded strain sensors. In the first case, the adhesive layer thickness and mechanical properties of this layer have a certain influence on the strain transferred from the structure to the bonded FBG [83]. Moreover, when bonded on a thin and low-modulus substrate, the FBG could change the original strain of the substrate [84]. Wan *et al.* [85] found that for a surface bonded fibre, the strain transfer is dominated by: (i) adhesive thickness between the bottom of the fibre and the substrate and (ii) the bond length of the fibre. Since embedded fibres are completely surrounded by the host material, one cannot speak of bond thickness and bond length in the second case. In this matter, apparent strain gradients only exist over ∼2 mm (depending on the material properties) starting at the entry point of the embedded fibre [86].

Several authors have embedded optical fibre Bragg sensors in composite specimens to measure axial strain. In the meantime, the relationship between the axial strain of the sensor and host material is discussed. Cox [87] analytically determined the uni-axial strain transfer for the case of a finite length cylindrical inclusion embedded into an isotropic material subjected to an axially constant strain field. This theory is often referred to as the shear-lag analysis, because the axial strain of the host material is transferred through the shear strain in the interface coating/optical fibre. However, this theory does not consider the mechanical properties of the host material. Afterwards, several authors improved the shear-lag theory. For example, Jiang *et al.* [88] developed formulas to predict the strain field distributions of fibre and host material by combining the shear-lag theory and the theory of elasticity. In 2009, Li *et al.* [89] gave an overview of the parameters (mechanical properties of the coating and host material, and the gauge length of the FBG) which can affect the strain transfer. Anyhow, the above referred literature proves the necessity of *in-situ* calibration of the gauge factor of embedded optical fibre sensors.

In some cases *no coating* (recoating) at the location of the FBG is chosen in axial strain sensing applications. As such, the strain acts directly onto the cladding’s surface of the optical fibre sensor. Fan *et al.* [90] determined experimentally the influence of the additionally induced radial strain in optical fibres when embedding them in a host material. The authors tried to quantify this mismatch by producing two [0_8_,90_4_]_s_ composite specimens in which two FBGs were embedded; one along the 0 degree direction and one along the 90 degree direction. Measurement errors up to 8% were found when using the bare FBGs gauge factor for axial strain. To correct this gauge factor, the authors suggested to use a coefficient which is dependent on the type of load, +3% for tensile loads and −8% for compressive loads. Care should, however, be taken by introducing correction coefficients which are dependent on the type or direction of the load.

#### 3.2.2. Multi-Axial Strain Sensing Applications

Nevertheless, direct strain transfer of the host material to the a optical fibre sensor without coating can be interesting in terms of *multi-axial strain measurements* of composite laminates. Former research activities by other authors [91,92] pointed out the need to determine the total strain field response of an embedded FBG in a host material. Bosia *et al.* [45] studied the response of a sensor embedded in an epoxy specimen experiencing biaxial loading. Finite elements showed the stress distribution in the vicinity of the embedded fibre. The difference in transverse strain of the host material and embedded sensor was evaluated for transversally applied loads. It proves the necessity of a multi-axial strain transfer approach in such situations. Prabhugoud and Peters [93] predicted the spectral response of an (embedded) FBG under a multi-axial strain field through a combined opto-mechanical study. Even the FBG spectral response for non-uniform strain and stress in the laminate could be predicted. The reverse problem, however, was not tackled. Kollar and Vansteenkiste [94] developed an analytical model of the multi-axial strain transfer between an embedded optical fibre and an infinite anisotropic host material. The stress and strain relationships where derived for loading and boundary conditions applied at infinity. However, composites can be relatively thin and the lay-up of the laminate and the position of the sensor in a certain layer should be taken into account to improve multi-axial measurements with optical fibres [40,50]. At the moment, an experimental approach is still non existing, although, the author believes that multi-axial strain measurements can be of added value in monitoring the structural integrity of composites.

It should be mentioned that the risk of fibre breakage when handling a stripped fibre is high, certainly with respect to embedding of the FBG. By using coated fibre sensors this risk can be diminished.

## 4. Temperature Compensation

The sections above have elaborated mainly on the effect on the FBG-spectrum of homogenous and non-homogenous strains, and on how this affects the method of measuring the Bragg peak wavelength shift, and on the influence of the strain transfer (longitudinal as well as transverse) with respect to the measured strain field with the optical fibre sensor, considering an atmosphere with constant temperature. However, since for FBGs a *cross-sensitivity for the strain and temperature* exists, one has to find solutions to compensate the strain for temperature effects. Several methods are studied in literature, a short overview is given below. For each solution, one can check if it is suitable for embedded strain sensing applications.

### 4.1. Extra Strain-Free Reference (FBG) Sensor

The most simple method is measuring the temperature with an *external temperature sensor* and then using it to compensate the intrinsic temperature effects of the embedded grating. A reference FBG can, for example, be used as an external temperature sensor. An extra *strain free FBG* is added in the measurement system [95–97] and discrimination between strain and temperature can be done by separately evaluating the Bragg wavelength shifts of both FBGs (Δ*λ*_*B*,1_,Δ*λ*_*B*,2_) with their respective strain and temperature sensitivity (*k_ε_*,*k_T_*):

(6) $$\left[\begin{array}{c} {\Delta \lambda }_{B,1}\\ {\Delta \lambda }_{B,2} \end{array}\right]=\left[\begin{array}{cc} k_{\varepsilon } & k_{T,1}\\ 0 & k_{T,2} \end{array}\right]\left[\begin{array}{c} \Delta \varepsilon \\ \Delta T \end{array}\right]$$

**Figure 9.**
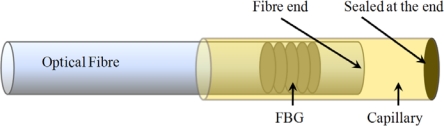
Schematic of an optical fibre Bragg grating embedded in a capillary to exclude all external stress/strain components.

Although not necessary for temperature compensation, the use of an identical FBG makes compensation very easy by just subtracting the wavelength shift of the strain free sensor from the total wavelength shift of the strain sensor. For embedded strain sensors this method can be expanded by embedding an extra Bragg sensor in a strain free compensating plate which has an identical lay-up as the structure in which the strain sensor is embedded [98]. As such, one can focus only on strain fields caused by external loads. The reference FBG should be located in the same thermal environment as the strain sensor. If one only wants to compensate for the intrinsic temperature behaviour of the grating, one can think of encapsulating a reference grating and embed this in the material. This reference grating should be isolated from the existing strain field in the material by ending it strain–free inside the capillary (glass, fused silica or metal) (Figure 9) [60,99–102].

### 4.2. Extra FBG Imposed on a Different Strain Field

Instead of a strain free configuration, one can also think of the use of an extra grating, *imposed on a different strain field* than the first grating. Since both gratings are then sensitive to strain and temperature, the discrimination method of Equation (6) should be extended with extra strain and temperature coefficients:

(7) $$\left[\begin{array}{c} {\Delta \lambda }_{B,1}\\ {\Delta \lambda }_{B,2} \end{array}\right]=\left[\begin{array}{cc} k_{\varepsilon ,1} & k_{T,1}\\ k_{\varepsilon ,2} & k_{T,2} \end{array}\right]\;\left[\begin{array}{c} \Delta \varepsilon \\ \Delta T \end{array}\right]$$

For example, when both gratings are loaded similarly, but opposite in sign (e.g., like in a bending experiment), temperature is compensated by just subtracting both wavelength shifts and dividing the result by two [103–105]. By artificially enlarging the stiffness of the compensating grating, it will sense a different strain (by integrating it in a capillary [101], by bonding it to another dummy fibre [106], or embedding it into another material [107]). Another special configuration is introduced by Silva *et al.* [108]. A transversal load sensor is created by winding a grating which is written in a polarization maintaining fibre, around a classical single mode fibre with grating so both gratings will react differently to transverse load, and temperature can be discriminated. James *et al.* [109] proposed a sensor configuration with an extra grating written in an optical fibre with a smaller diameter spliced to the first FBG (strain sensor). The same effect can be reached by etching the fibre at the grating position so that it becomes smaller [110]. However, it should be mentioned that, these configurations are prone to breaking during the embedding process and lose all of their efficiency (like any of the extra grating configuration discussed before) when considering the embedded situation.

### 4.3. Extra FBGs Which React Differently to the Same Strain Field

Another technique is adding an extra grating which *reacts differently to the same strain field*. This can be done by using different FBG types which have different temperature-induced wavelength shifts [111–114], or by using a second grating which is differently doped than the first one [115–118]. A lot of research has been done on the fabrication of hybrid sensors which combine an FBG with another (‘similar’) type of sensor [119–122]. These last configurations all have in common that the demodulation of the sensor signal becomes a lot more complicated. In the last decade, researchers have also focused on discrimination of strain and temperature using Bragg gratings written in polarization maintaining (PM) and micro-structured fibres. Although a small difference exists in temperature sensitivity [48,123,124] for the Bragg peaks of FBGs written in classical PM-fibres (bow-tie, panda, and elliptical core), discriminating axial strain and temperature remains very difficult [44,60]. In classical PM-fibre, birefringence is most of the time induced by temperature dependent stress applying parts while in micro-structured fibre, this mainly originates from the micro-structure inside the fibre. Therefore, almost no difference exists in temperature sensitivity for both polarization axes of the micro-structure fibre. By subtracting both wavelength shifts, one creates a temperature-independent measurement [125,126].

Note that in most of the discussed configurations a discrimination is made between temperature and axial strain. The user should, however, be careful when the fibre Bragg grating is embedded, for example in reinforced plastics, that transversal strains act in a different way on the grating than when not embedded. In addition, note that if a second order polynomial temperature effect is considered (Section 2) which is definitely necessary for cure monitoring purposes, only the reference temperature sensor method can be used (Section 4.1).

## 5. Conclusions

This review paper has given an overview on how to measure strains in composite materials, using embedded fibre Bragg gratings. Although it is clear that FBGs, thanks to their compatibility with fibrous composite materials, are the best choice for embedded strain monitoring of composite materials, some *side remarks* were given on the difficulties to implement them in a controlled and automated manner (e.g., the *fibre entry point)*. The strain and temperature sensitivity were briefly discussed. Attention was drawn to the *non-linearity* of the Bragg wavelength shift as a function of temperature for *high temperature ranges*.

Then, *a monitoring scheme* was introduced, which showed the different steps necessary to relate the output of an embedded FBG with the strain of the structure in which it is embedded. The first step is about the *calibration of non-embedded FBGs*. The longitudinal strain calibration is more or less straightforward. However, it should be mentioned that, when being embedded, the FBG is subjected to transverse strains (in-plane and out-of-plane) as well. Therefore, an FBG needs to be calibrated for transverse strains. Research has shown that transverse strain calibrations are difficult to control and that the wavelength shifts are difficult to relate to the induced strains, certainly in the case of high birefringent fibres. New and controlled techniques are necessary to perform a correct transverse strain calibration, which can relate the applied load to the strains in embedded FBGs. It has been shown that the back-reflected light spectrum of an FBG tends to distort during the curing process. The shape of the distortion depends on the type of composite laminate (UD, crossply, *etc*.) in which it has been embedded. Consequently, different *Bragg wavelength determination methods* are necessary. A thorough and complete overview of available methods can be found in the literature. Once the strain of the optical fibre is determined, it should be related to the strain of the composite structure. In this matter, it should not be forgotten that embedded FBGs are subjected not only to longitudinal strains but to the complete internal strain field of the structure!

Last but not least, some useful *temperature compensation techniques* for embedded FBG strain monitoring are discussed. A conclusion is that, if the non-linearity of the wavelength shift as function of temperature is considered (Section 2), the only possible method is the reference temperature sensor method. It should be clear from this review paper that a lot of effort has gone into to making FBG-sensors the best to measure internal strains of composite structures. However, despite the enormous effort, several difficulties remain for research to overcome.
